# Supplementary material for: A versatile clearing agent for multi-modal brain imaging
Source: Sci Rep. 2015 May 7;5:9808. doi: 10.1038/srep09808 (PMC4423470; doi:10.1038/srep09808)
Supplement: Supplementary Information [file srep09808-s1.pdf]

A versatile clearing agent for multi modal brain imaging

Irene Costantini[1], Jean-Pierre Ghobril[4], Antonino Paolo Di Giovanna[1], Anna Letizia Allegra Mascaro[1], Ludovico Silvestri[1], Marie Caroline Müllenbroich[1], Leonardo Onofri[1], Valerio Conti[6], Francesco Vanzi[1,7], Leonardo Sacconi[2,1], Renzo Guerrini[6], Henry Markram[4], Giulio Iannello[5], Francesco Saverio Pavone[1,2,3*]

1. European Laboratory for Non-linear Spectroscopy, University of Florence, Via Nello Carrara 1, 50019 Sesto Fiorentino, Italy
2. National Institute of Optics, National Research Council, Largo Fermi 6, 50125 Florence, Italy
3. Department of Physics and Astronomy, University of Florence, Via Sansone 1, 50019 Sesto Fiorentino, Italy
4. Laboratory of Neural Microcircuitry, Brain Mind Institute, EPFL, Station 15, CH-1015 Lausanne, Switzerland
5. Department of Engineering, University Campus Bio-Medico of Rome, Via Alvaro del Portillo 21, 00128 Roma, Italy
6. Pediatric Neurology and Neurogenetics Unit and Laboratories, Department of Neuroscience, Pharmacology and Child Health, A. Meyer Children's Hospital - University of Florence, Viale Pieraccini 24, 50139 Florence, Italy
7. Department of Biology, University of Florence, Via Romana 17, 50125 Florence, Italy

*corresponding author email: francesco.pavone@unifi.it

Supplementary Figure 1

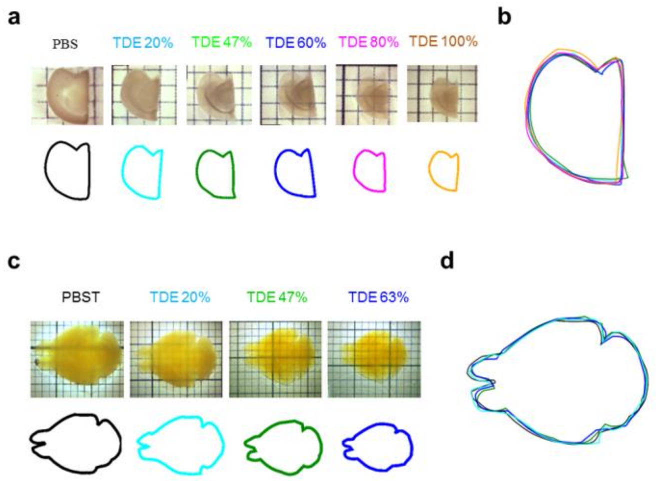

Supplementary Figure 1. TDE induces an homogeneous brain tissue shrinkage. (a) Transmission images and edges tracing of 1 mm thick hemi-brain slices in PBS and after clearing with various solutions. (b) Superimposed image of the edges resized using the linear deformation parameter obtained in figure 1. (c) Transmission images and edges tracing of a mouse brain after CLARITY protocol. (d) Superimposed image of the edges resized using the linear deformation parameter obtained in figure 3.

Supplementary Figure 2

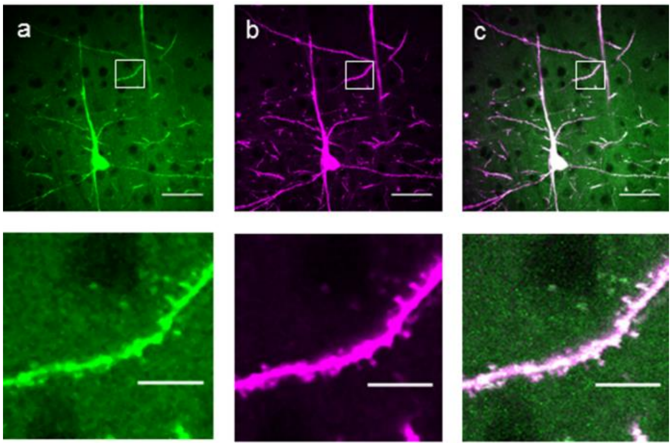

Supplementary Figure 2. Mouse brain immunostaining. 1 mm Thy1-GFP-M mouse brain slice perfused with PFA and subsequently treated with passive CLARITY (PC), immunostained with an anti-GFP IgG alexa fluor 594 conjugate and cleared with 47% TDE/PBS. (a) GFP (green); (b) Ig anti-GFP (magenta); (c) overlay. Scale bar = 50 µm (upper panels) and 10 µm (lower panels).

Supplementary Figure 3

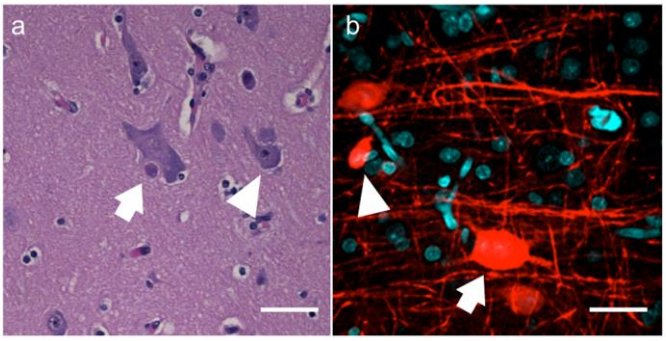

Supplementary Figure 3. Human brain histology. (a) Hematoxylin/eosin staining of paraffin embedded tissue and (b) Two-photon imaging of the sample treated with CLARITY protocol (passive clearing), cleared with TDE and immunostained with anti-parvalbumin (in red) and nuclei marker (DAPI, in cyan) demonstrating that the features observed in the cleared tissue are directly comparable to those obtained with conventional staining techniques. White arrows indicate giant dysmorphic neurons, white arrowheads indicate wild-type neurons. Scale bar = 50 µm.

Supplementary Figure 4

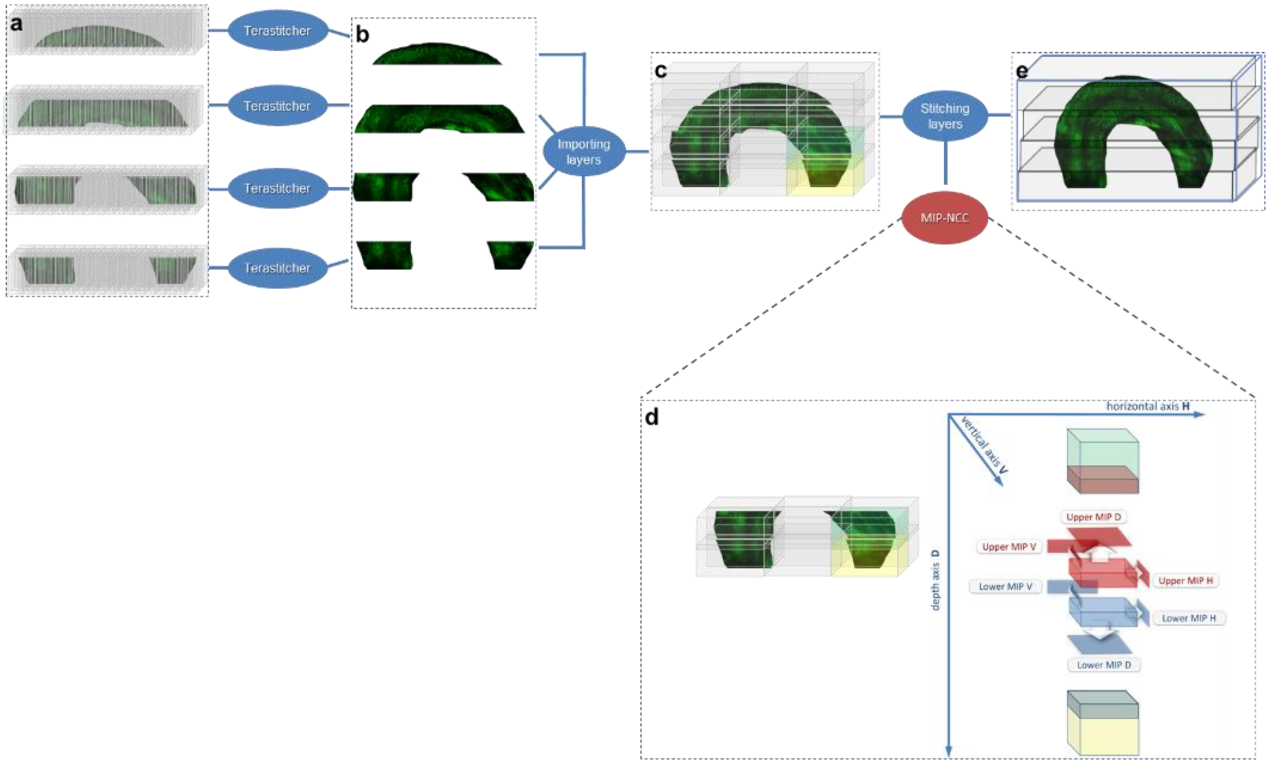

Supplementary Figure 4. Stitching processing pipeline. From left to right: (a) Various input layers composed of overlapping stacks are separately stitched by using the Terastitcher. (b,c) The stitched layers are imported into a volume with overlapping regions between layers. (d) Displacements for each pair of adjacent layers are computed. (e) Different layers are merged into a single 3D image
